# Assessment of Inadequate Use of Pediatric Emergency Medical Transport Services: The Pediatric Emergency and Ambulance Critical Evaluation (PEACE) Study

**DOI:** 10.3389/fped.2019.00442

**Published:** 2019-10-25

**Authors:** Martin Poryo, Martin Burger, Stefan Wagenpfeil, Bennet Ziegler, Harald Sauer, Marina Flotats-Bastardas, Ulrich Grundmann, Michael Zemlin, Sascha Meyer

**Affiliations:** ^1^Department of Pediatric Cardiology, Saarland University Medical Center, Homburg, Germany; ^2^Medical School, University of Saarland, Homburg, Germany; ^3^Institute for Medical Biometry, Epidemiology and Medical Informatics, Saarland University Medical Center, Homburg, Germany; ^4^SPG, Saarpfalz-Gymnasium, Homburg, Germany; ^5^Department of Neuropediatrics, Saarland University Medical Center, Homburg, Germany; ^6^Department of Anesthesiology, Intensive Care and Pain Therapy, Saarland University Medical Center, Homburg, Germany; ^7^Department of Pediatrics and Neonatology, Saarland University Medical Center, Homburg, Germany

**Keywords:** ambulance, emergency medical transport service, misuse, pediatric emergency, public health

## Abstract

**Aim:** To provide data on the inadequate use of emergency medical transports services (EMTS) in children and underlying contributing factors.

**Methods:** This was a prospective single-center cohort study (01/2017-12/2017) performed at the Saarland University Children's Hospital, Homburg, Germany. Patients ≤20 years of age transported by EMTS for suspected acute illness/trauma were included and proportion of inadequate/adequate EMTS use, underlying contributing factors, and additional costs were analyzed.

**Results:** Three hundred seventy-nine patients (mean age: 9.0 ± 6.3 years; 55.7% male, 44.3% female) were included in this study. The three most common reasons for EMTS use were: central nervous system (30.6%), respiratory system affection (14.0%), and traumas (13.2%). ETMS use was categorized as inadequate depending on physician's experience: senior physician (58.8%), pediatrician (54.9%), resident (52.7%). All three physicians considered 127 (33.5%) cases to be medically indicated for transportation by EMTS, and 177 (46.7%) to be medically not indicated. The following parameters were significantly associated with inadequate EMTS use: non-acute onset of symptoms (OR 2.5), parental perception as non-life-threatening (OR 1.7), and subsequent out-patient treatment (OR 4.0). Conversely, transport by an emergency physician (OR 3.5) and first time parental EMTS call (OR 1.7) were associated with adequate use of EMTS. Moreover, a significant relation existed between maternal, respectively, paternal educational status and inadequate EMTS use (each *p* = 0.01). Using multiple logistic regression analysis, non-acute onset of symptoms (OR 2.2) was associated with inadequate use of EMTS while first time parental EMTS call (OR 1.8), transport by an emergency physician (OR 3.3), and need for in-patient treatment (OR 4.0) were associated with adequate use of EMTS.

**Conclusion:** A substantial number of pediatric EMTS is medically not indicated. Possibly, specific measures including multifaceted educational efforts may be helpful in reducing unnecessary EMTS use.

## Introduction

Overcrowded emergency departments (ED) are a well-known problem in highly-industrialized countries (1, 2). One important reason is the increasing demand for hospital and ED care, but the misuse of emergency medical transport services (EMTS) as a taxi service must be taken into consideration as well. Interestingly, the incidence of life-threatening illness in children is at an all-time low while presentations and admissions to hospital increase (3). Several studies have demonstrated that up to 61% of EMTS are not medically necessary (4–10), thus putting a relevant financial burden on the health-care system.

While pediatric emergencies are rare at an estimated rate of 5–10% of all EMTS (11–14), misuse of the EMTS has been reported for children as well (8, 9, 15). Among a number of contributing factors, parental over-anxiety appears to be one of the most relevant reasons (16). Conversely, rapid assessment of the medical necessity whether or not to use an EMTS in children is challenging for parents and teachers as well as EMTS staff, in part because of poor medical routine in pediatric emergencies.

The aim of this study was to provide current data on the inadequate EMTS use in children in Germany (Saarpfalz region). Secondly, we performed an explorative analysis of potential, underlying factors that may contribute to this problem. Thirdly, an estimation of additional costs secondary to the unnecessary EMTS use was made.

## Patients and Methods

After institutional review board approval from the ethics committee of Saarland, Saarbrücken, Germany, this prospective explorative study (PEACE study) was performed at the University Children's Hospital of Saarland, Homburg/Saar, Germany (Saarpfalz region) between 01 January and 31 December 2017. The PEACE study was reported using the STROBE guideline (Supplementary Data Sheet 1) (17).

The pediatric emergency department of the Saarland University Medical Center, Homburg/Saar, Germany, is the largest emergency department in Saarland with ~5,000 visits per year.

The time period of 1 year was considered representative since acute diseases typically occurring at different time points (seasons) were included in this study, generating a convenient and adequate sample size of children using EMTS.

All patients ≤20 years of age who were transported to our ED by EMTS were assessed for potential enrollment in this study. The upper limit of age of ≤20 years was attributed to the fact that patients with complex, pediatric diseases were included. These patients are primarily transferred to our children's hospital by EMTS because of ongoing treatment of these patients in our hospital, e.g., children/adolescents/young adults with complex congenital heart disease, and patients with complex genetic syndromes affecting the central nervous system. Exclusion criteria were: lack of parental informed consent and transfer from another hospital/doctor's office per EMTS, and patients requiring immediate resuscitation, or patient-specific missing data >10%.

Patients' data were collected prospectively from the hand-written and electronic patients' medical charts (SAP, Walldorf, Germany) and included the following information: gender, age, transport by EMTS only or staffed with an emergency physician, suspected diagnosis at admission, time, week-day and month of presentation. Potential underlying risk factors as detailed below were specified *a priori*.

All participating families were asked to fill out a questionnaire, which included questions with regard to the current and past medical problems and the social status of the family. The following questions were chosen for data analysis:

Questions regarding the emergency:

1. Duration of symptoms: acute = sudden onset of symptoms, non-acute = duration of symptoms for several hours or longer
2. Perception of situation as life-threatening?
3. First-time call of EMTS for your child?
4. If “no,” how often have you used EMTS?
5. Availability of car or access to other transport possibilities (e.g., public transport)?

Questions regarding parental/family status

1. Number of children? Single parent?
2. Parental educational status: low = no graduation, special-needs school, secondary school or middle = middle school, academic high school or higher = university
3. Current occupation: unskilled, skilled, highly skilled, others
4. Parental age: <40 years, 40–60 years, >60 years.

All included cases were evaluated by a senior physician in pediatrics and pediatric emergency medicine (SM), a fully trained pediatrician (MP), and a resident (MB). All three physicians were blinded to each other.

The included medical cases were categorized down into:

1. Yes, EMTS use was medically indicated.
2. No, EMTS use was medically not indicated, including EMTS medically not indicated but considered reasonable because of specific circumstances (e.g., patient at school and transportation by car not feasible).
3. Assessment was not possible because of inadequate medical information.

For final statistical analysis, the assessment by the senior physician was used because his long-standing experience was considered most reliable in providing a correct medical assessment. Inter-rater agreement was calculated using Cohen's Kappa coefficient.

“Medically indicated” was defined as “A sudden and usually unforeseen event that calls for immediate measures to minimize its adverse consequences” (18, 19). Immediate means that 95% of the emergency patients have to be seen by EMTS within 20 min of receipt of emergency call. At time of assessment, all three physicians were unaware of the further medical course (e.g., need for hospital admission etc.) of included patients.

“Medically not indicated” was defined as any clinical condition that did not mandate immediate medical assessment, diagnostic work-up, or treatment.

In the *federal state of* Saarland, costs for EMTS operations during the study period were [https://www.zrf-saar.de/de/wir_ueber_uns/aufgaben_des_zrf_saar/finanzierung_tarifverhandlungen (accessed 28.12.2019)]:

- 441.00 € for transport by EMTS only
- 870.00 € for transport by accompanying emergency physician.

Data were pseudonymized for statistical analysis. Statistical analysis was performed using IBM SPSS Statistics (IBM Corp., Released 2010; IBM SPSS Statistics for Macintosh, Version 19, Armonk, NY, USA). Data is presented as absolute numbers, percentage, odds ratios (OR), and 95% confidence intervals (95%-CI). Binary logistic regression analysis was computed with forward and backward (Wald) method. In cases of incongruent results, only the results of the backward computed logistic regression are reported. For each variable, univariate logistic regression analysis was performed, and all significant variables were included into the multiple logistic regression analysis. Since the aim of the regression analysis was to identify variables which contributed to the adequate/inadequate EMTS use, the “adequate EMTS use,” respectively, the “inadequate EMTS use” were the dependent variables. The independent variables were the potential, underlying factors that may have contributed to this problem. Comparison between variables was performed using *Chi*^2^*-test*. A *p*-value of <0.05 was considered statistically significant.

To explore a potential bias, both data from patients with parental consent as well as data where parental consent could not be obtained for the questionnaire were included, and we subsequently analyzed the basic data with regard to demographics and assessment of the adequacy of EMTS use of the two groups (Supplementary Table 1).

## Results

**Figure 1:**
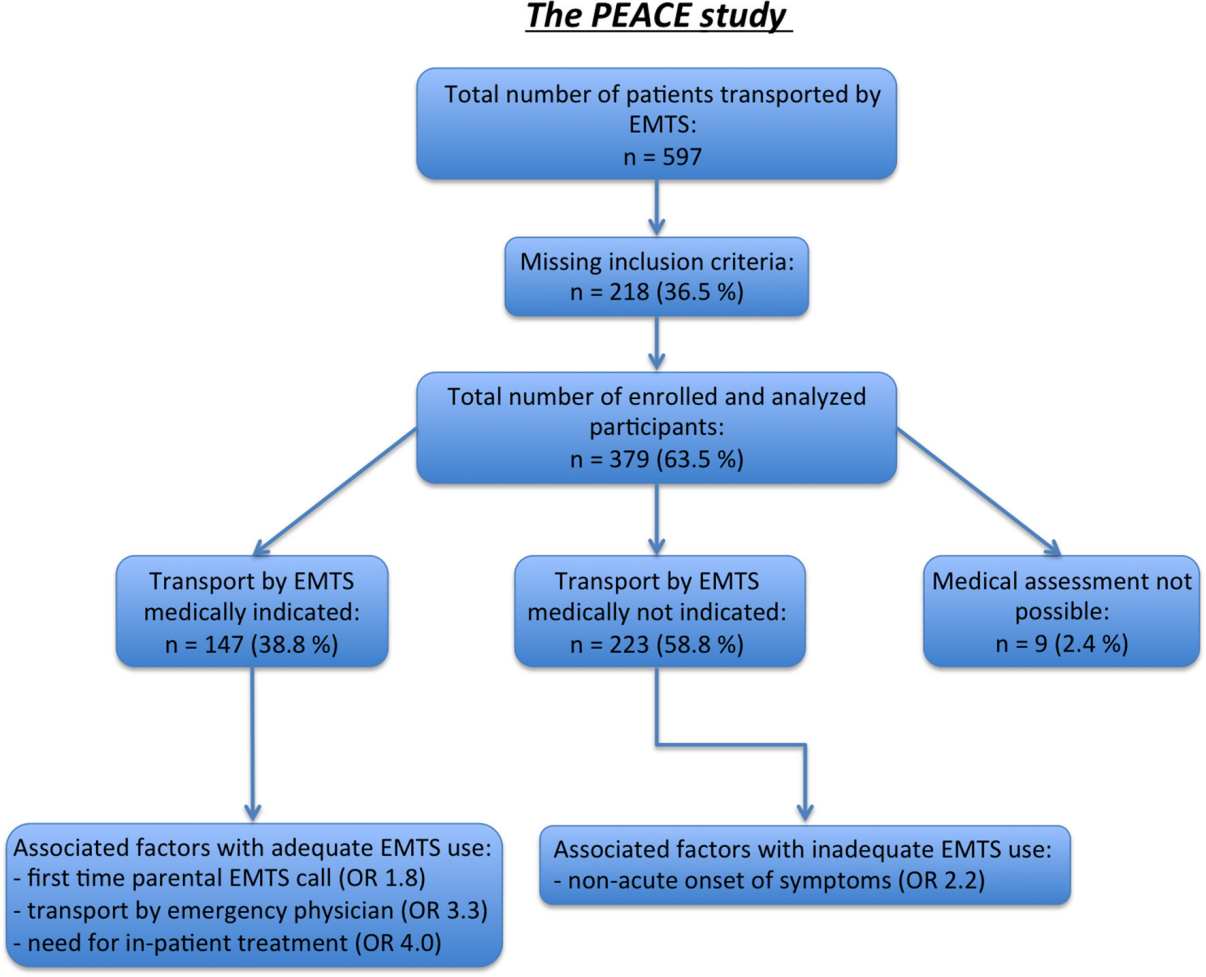
STROBE flowchart.

In total, 597 pediatric patients brought to our hospital by EMTS from home or non-medical facilities were assessed. After exclusion of patients who did not meet the inclusion criteria (most common cause: lack of parental consent) 379 patients (mean age: 9.0 ± 6.3 years, range: 0–19.2 years; 55.7% male, 44.3% female) were enrolled and subsequently analyzed (Figure 1). Patients' and family characteristics are depicted in **Tables 4**, **5**.

In most cases (*n* = 330, 87.1%), parents did not contact their family physician, family general physician or a hospital. The three most common reasons for EMTS use were: central nervous system (CNS) (*n* = 116, 30.6%) and pulmonary system affection (*n* = 53, 14.0%), and traumas (*n* = 50, 13.2%). An analysis of the affected organ systems by age is depicted in Table 1.

**Table 1:** Affected organ system at admission by ambulance categorized by age.

**Affected organ system**	**≤28 days (*n* = 2)**	**28 days−1 year (*n* = 43)**	**1–12 years (*n* = 186)**	**13–20 years (*n* = 148)**
Central nervous system	0 (0.0%)	7 (16.3%)	65 (34.9%)	44 (29.7%)
Pulmonary	0 (0.0%)	7 (16.3%)	35 (18.8%)	11 (7.4%)
Cardiac	0 (0.0%)	1 (2.3%)	13 (7.0%)	35 (23.6%)
Gastrointestinal	1 (50.0%)	5 (11.6%)	24 (12.9%)	15 (10.1%)
Trauma	1 (50.0%)	14 (32.6%)	32 (17.2%)	3 (2.0%)
Intoxication	0 (0.0%)	2 (4.7%)	8 (4.3%)	29 (19.6%)
Others	0 (0.0%)	7 (16.3%)	9 (4.8%)	11 (7.4%)

*Data are presented as absolute numbers and percentages (brackets). Percentage refers to absolute numbers within the same line*.

All three physicians considered 127 (33.5%) cases to be medically indicated for transportation by EMTS, and 177 (46.7%) not to be medically indicated. In 75 (19.8%) cases, all three physicians could not agree on the necessity for EMTS. The assessment of inadequate EMTS was depending on the physician's experience: senior physician in pediatrics and emergency medicine: 58.8%; 13.2% of EMTS were medically not indicated, but considered reasonable because of specific circumstances; pediatrician: 54.9%; 20.3% of EMTS medically not indicated, but considered reasonable because of specific circumstances; resident: 52.7%; 12.1% medically not indicated, but considered reasonable because of specific circumstances (Table 2).

**Table 2:** Evaluation of the medical necessity for EMTS (emergency medical transport service).

**Evaluating physicians**	**Medically indicated**	**Medically not indicated**	**Medical assessment not possible**
Chief resident in pediatrics and pediatric emergency medicine (*n* = 379)	147 (38.8%)	223 (58.8%)	9 (2.4%)
Pediatrician (*n* = 379)	161 (42.5%)	208 (54.9%)	10 (2.6%)
Resident (*n* = 379)	172 (45.5%)	200 (52.7%)	7 (1.8%)

*Data are presented as absolute number and percentages (brackets). Percentage refers to absolute numbers within the same line*.

The inter-rater agreement was as followed: rater 1 vs. rater 2: Kappa 0.777, *p* = 0.000, rater 1 vs. rater 3: Kappa 0.747, *p* = 0.000 and rater 2 vs. rater 3: Kappa 0.770, *p* = 0.000.

In the group of adequate EMTS, the three most common indications were: CNS affection (*n* = 56, 38.1%), intoxications (*n* = 25, 17.0%), and pulmonary disorders (*n* = 22, 15.0%). In the group of inadequate EMTS, the three most common indications were: CNS (*n* = 58, 26.0%), gastrointestinal (*n* = 40, 17.9%), and the cardiovascular system (*n* = 33, 14.8%). The number of non-acute complaints in the group of inadequate EMTS was almost twice as high as in the group of adequate EMTS (Table 3).

**Table 3:** Duration of symptoms categorized by medically indicated, respectively, not indicated EMTS (emergency medical transport service).

**Duration of symptoms**	**Medically** **indicated** **(*n* = 147)**	**Medically not** **indicated** **(*n* = 223)**
Acute	126 (85.7%)	158 (70.9%)
For several hours	11 (7.5%)	31 (13.9%)
For several days	8 (5.4%)	24 (10.8%)
For several weeks	2 (1.4%)	10 (4.5%)

*Data are presented as absolute numbers and percentages (brackets). Percentage refers to absolute numbers within the same group; in 9 patients definite medical assessment not possible*.

The following parameters were significantly associated with inadequate EMTS use (Tables 4, 5): non-acute onset of symptoms, parental perception as non-life-threatening, and subsequent out-patient treatment. Conversely, transport by an emergency physician and first time parental EMTS-call were associated with adequate use of EMTS. Moreover, a significant relation existed between maternal, respectively, paternal educational status and inadequate EMTS use (each *p* = 0.01).

**Table 4:** Patients' characteristics.

**Patients' characteristics and EMTS specifics**	**Overall** **(*n =* 379)**	Medically Indicated^*^ (*n =* 147)	Medically not Indicated^*^ (*n =* 223)	**Definite medical** **evaluation** **not possible** **(*n =* 9)**	**OR**	**95 %-CI**	p-value^*^
Gender^*^							
Male	211 (55.7 %)	86 (58.5 %)	121 (54.3 %)	4 (44.4 %)	1.2	0.78–1.81	0.42
Female	168 (44.3 %)	61 (41.5 %)	102 (45.7 %)	5 (55.6 %)			
**Age**^**#**^							
≤28 days	2 (0.5 %)	0 (0.0 %)	2 (0.9 %)	0 (0.0 %)			0.42
29 days−1 year	43 (11.3 %)	17 (11.6 %)	25 (11.2 %)	1 (11.1 %)			
1–12 years	186 (49.1 %)	79 (53.7 %)	104 (46.6 %)	3 (33.3 %)			
13–20 years	148 (39.1 %)	51 (34.7 %)	92 (41.3 %)	5 (55.6 %)			
Type of referral^*^							
Ambulance	279 (73.6 %)	86 (58.5 %)	185 (83.0 %)	8 (88.9 %)	**3.5**	**2.13**–**5.56**	**0**
Emergency physician	100 (26.4 %)	61 (41.5 %)	38 (17.0 %)	1 (11.1 %)			
Type of treatment^*^							
Out-patient	136 (35.9 %)	27 (18.4 %)	105 (47.1 %)	4 (44.4 %)	**4**	**2.43**–**6.67**	**0**
In-patient	243 (64.1 %)	120 (81.6 %)	118 (52.9 %)	5 (55.6 %)			
**Time of presentation**							
8 a.m.−4 p.m.	171 (45.1 %)	64 (43.5 %)	107 (48.0 %)	0 (0.0 %)			0.43
4–22 p.m.	122 (32.2 %)	45 (30.6 %)	71 (31.8 %)	6 (66.7 %)			
22 p.m.−8 a.m.	86 (22.7 %)	38 (25.9 %)	45 (20.2 %)	3 (33.3 %)			
Weekday of presentation^*^							
Workday	254 (67.0 %)	95 (64.6 %)	154 (69.1 %)	5 (55.6 %)	1.2	0.79–1.89	0.37
Weekend	125 (33.0 %)	52 (35.4 %)	69 (30.9 %)	4 (44.4 %)			
**Season**^**#**^							
January–March	142 (37.5 %)	43 (29.3 %)	94 (42.2 %)	5 (55.6 %)			0.1
April–June	78 (20.6 %)	35 (23.8 %)	43 (19.3 %)	0 (0.0 %)			
July–September	67 (17.7 %)	30 (20.4 %)	36 (16.1 %)	1 (11.1 %)			
October–December	92 (24.3 %)	39 (26.5 %)	50 (22.4 %)	3 (33.3 %)			
Child with chronic medical condition^*^							
Yes	120 (31.7 %)	48 (32.7 %)	70 (31.4 %)	2 (22.2. %)	1.1	0.68–1.66	0.8
No	259 (68.3 %)	99 (67.3 %)	153 (68.6 %)	7 (77.8 %)			
Child with long-term medication^*^							
Yes	93 (24.5 %)	39 (26.5 %)	54 (24.2 %)	0 (0.0 %)	1.1	0.70–1.82	0.62
No	286 (75.5 %)	108 (73.5 %)	169 (75.8 %)	9 (100 %)			
**Duration of symptoms**							
Acute	293 (77.3 %)	126 (85.7 %)	158 (70.9 %)	9 (100 %)	**2.5**	**1.43**–**4.26**	**0.001**
Non-acute	86 (22.7 %)	21 (14.3 %)	65 (29.1 %)	0 (0.0 %)			
Receiving medical treatment because of current complaints^*^							
Yes	136 (35.9 %)	45 (30.6 %)	90 (40.4 %)	1 (11.1 %)	1.5	0.99–2.38	0.06
No	243 (64.1 %)	102 (69.4 %)	133 (59.6 %)	8 (88.9 %)			
Doctor's appointment because of current complaints^*^							
Yes	29 (7.7 %)	12 (8.2 %)	17 (7.6 %)	0 (0.0 %)	1.1	0.25–2.33	0.85
No	350 (92.3 %)	135 (91.8 %)	206 (92.4 %)	9 (100 %)			
**Localization at onset of symptoms**^**#**^							
Private setting	220 (58.0 %)	91 (61.9 %)	123 (55.2 %)	6 (66.7 %)			0.3
Public setting	79 (20.8 %)	30 (20.4 %)	46 (20.6 %)	3 (33.3 %)			
Kindergarten/school	80 (21.1 %)	26 (17.7 %)	54 (24.2 %)	0 (0.0 %)			

*Data are presented as absolute number and percentages (brackets). Percentage refers to absolute numbers within the same group*.

*OR, odds ratio*.

*95%-CI = 95%-confidence interval*.

* *Binary logistic regression analysis was computed with the forward and backward method (Wald). In cases of conflicting results between the two methods, only results from backward computed logistic regression are reported*.

# *Fisher's exact test if one of the expected cell frequencies was <5; Chi^2^ test if all the expected cell frequencies were ≥5*.

*p-values <0.05 were considered statistical significant*.

*Significant values are indicated in bold*.

**Table 5:** Social characteristics of patients' families.

**Social characteristics and parental perceptions, experience and knowledge with regard to EMTS**	**Overall** **(*n =* 379)**	**Medically** indicated^*^ (*n =* 147)	**Medically not** indicated^*^ (*n =* 223)	**Definite medical evaluation** **not possible** **(*n =* 9)**	**OR**	**95%-CI**	**p-value**
Number of children^*^							
1	107 (28.2%)	33 (22.4%)	71 (31.8%)	3 (33.3%)	1.6	0.99–2.56	0.06
≥2	271 (71.7%)	113 (76.9%)	152 (68.2%)	6 (66.7%)			
Single parent^*^							
Yes	100 (26.6%)	39 (26.7%)	60 (27.1%)	1 (11.1%)	1	0.61–1.57	0.93
No	276 (73.4%)	107 (73.3%)	161 (72.9%)	8 (88.9%)			
Access to other transport possibilities than ambulance^*^							
Yes	328 (86.8%)	132 (90.4%)	187 (83.9%)	9 (100%)	1.8	0.94–3.5	0.08
No	50 (13.2%)	14 (9.6%)	36 (16.1%)	0 (0.0%)			
Perception as life-threatening situation^*^							
Yes	170 (46.1%)	78 (53.4%)	88 (40.9%)	4 (50.0%)	**1.7**	**1.08**–**2.53**	**0.02**
No	199 (53.9%)	68 (46.6%)	127 (59.1%)	4 (50.0%)			
Perception child may die^*^							
Yes	351 (92.6%)	138 (93.9%)	205 (91.9%)	8 (88.9%)	1.3	0.59–3.08	0.48
No	28 (7.4%)	9 (6.1%)	18 (8.1%)	1 (11.1%)			
Parents called the rescue service for the first time because of their child^*^							
Yes	213 (56.3%)	95 (64.6%)	115 (51.8%)	3 (33.3%)	**1.7**	**1.11**–**2.61**	**0.02**
No	165 (43.7%)	52 (35.4%)	107 (48.2%)	6 (66.7%)			
**Total number of occasions parents used the emts**^**#**^							
2	93 (24.6%)	28 (19.0%)	62 (28.1%)	3 (33.3%)			0.08
3	25 (6.6%)	6 (4.1%)	18 (8.1%)	1 (11.1%)			
4	14 (3.7%)	4 (2.7%)	9 (4.1%)	1 (11.1%)			
>4	30 (7.9%)	13 (8.8%)	16 (7.2%)	1 (11.1%)			
**Parental efforts to get other form of medical help before calling EMTS**							
Yes	48 (12.7%)	21 (14.3%)	27 (12.2%)	0 (0.0%)	1.2	0.65–2.22	0.55
No	330 (87.3%)	126 (85.7%)	195 (87.8%)	9 (100%)			
**Maternal educational status**^**#**^							
Low	103 (31.3%)	28 (22.0%)	73 (37.4%)	2 (28.6%)			**0.01**
Middle	182 (55.3%)	78 (61.4%)	100 (51.3%)	4 (57.1%)			
High	44 (13.4%)	21 (16.5%)	22 (11.3%)	1 (14.3%)			
**Paternal educational status**^**#**^							
Low	113 (35.2%)	30 (24.6%)	81 (42.4%)	2 (25.0%)			**0.01**
Middle	153 (47.7%)	66 (54.1%)	82 (42.9%)	5 (62.5%)			
High	55 (17.1%)	26 (21.3%)	28 (14.7%)	1 (12.5%)			
**Current maternal occupation**^**#**^							
Unskilled	53 (14.0%)	19 (12.9%)	34 (15.2%)	0 (0.0%)			0.42
Skilled	207 (54.6%)	87 (59.2%)	114 (51.1%)	6 (66.7%)			
Highly skilled	26 (6.9%)	10 (6.8%)	14 (6.3%)	2 (22.2%)			
Others	93 (24.5%)	31 (21.1%)	61 (27.4%)	1 (11.1%)			
**Current paternal occupation**^**#**^							
Unskilled	53 (14.0%)	19 (12.9%)	34 (15.2%)	0 (0.0%)			0.46
Skilled	225 (59.4%)	93 (63.3%)	126 (56.5%)	6 (66.7%)			
Highly skilled	35 (9.2%)	14 (9.5%)	19 (8.5%)	2 (22.2%)			
Others	66 (17.4%)	21 (14.3%)	44 (19.7%)	1 (11.1%)			
**Maternal age**^**#**^							
<40 years	199 (53.2%)	82 (56.2%)	114 (52.1%)	3 (33.3%)			0.74
40–60 years	173 (46.3%)	63 (43.2%)	104 (47.5%)	6 (66.7%)			
>60 years	2 (0.5%)	1 (0.7%)	1 (0.5%)	0 (0.0%)			
**Paternal age**^**#**^							
<40 years	156 (42.6%)	60 (41.7%)	93 (43.5%)	3 (37.5%)	0.67		
40–60 years	201 (54.9%)	79 (54.9%)	117 (54.7%)	5 (62.5%)			
>60 years	9 (2.5%)	5 (3.5%)	4 (1.9%)	0 (0.0%)			

*Data are presented as absolute numbers and percentages (brackets). Percentage refers to absolute numbers within the same group*.

*OR, odds ratio*.

*95%-CI = 95%-confidence interval*.

* *Binary logistic regression analysis was computed with the forward and backward method (Wald). In cases of conflicting results between the two methods, only results from backward computed logistic regression are reported*.

# *Fisher's exact test if one of the expected cell frequencies was <5; Chi^2^ test if all the expected cell frequencies were ≥5*.

*p-values <0.05 were considered statistical significant*.

*Significant values are indicated in bold*.

Using multiple logistic regression analysis, non-acute onset of symptoms (OR 2.2, 95%-CI [1.20–3.87]) was associated with inadequate use of EMTS, while first time parental EMTS call (OR 1.8, 95%-CI [1.12–2.03]), transport by emergency physician (OR 3.3, 95%-CI [1.95–5.49]), and need for in-patient treatment (OR 4.0, 95%-CI [2.39–6.85]) were associated with adequate use of EMTS.

In total, medical expenditures of 210,039 € incurred in our cohort; 123,039 € related to EMTS use and 87,000 € related to EMTS use operated by emergency physicians. More than half of these costs was secondary to inadequate EMTS use, causing additional costs of 114,645 € (ambulance 81,585 € and emergency physician 33,060 €) per year.

## Discussion

In our prospective exploratory PEACE study, a substantial number of EMTS use was medically not indicated (46.7%). We were able to demonstrate that a number of underlying risk factors were contributory to the inadequate use of EMTS, i.e., non-acute onset of symptoms (OR 2.5), parental perception as non-life-threatening (OR 1.7), and subsequent out-patient treatment (OR 4.0), while transport by emergency physician (OR 3.5) and first time parental EMTS-call (OR 1.7) were associated with adequate use of EMTS. The physician's assessment with regard to the adequacy of EMTS use was dependent on the level of clinical experience of the evaluating physician, i.e. the more experienced the physician, the higher the rate of unnecessary use of EMTS.

Particularly, parental educational status [maternal (*p* = 0.01) and paternal educational status (*p* = 0.01)] had an important impact on the misuse of the EMTS. Interestingly, medium socio-economic status was associated with the lowest percentage of inadequate EMTS use. Although quite speculative, low socio-economic status may possibly be associated with poorer medical knowledge while over-anxiety regarding child's health may be more prevalent in higher socio-economic classes, thus contributing to the inadequate use of EMTS. Conversely, occupational status and parental age were not significantly associated with adequate/inadequate use of EMTS. Interestingly, data from the U.S. (8) demonstrated that the inadequate use of EMTS in children was strongly associated with their medical insurance status (unnecessary use occurred significantly more often in children with Medicaid insurance compared to commercial insurance coverage), indicating that socio-economic factors are contributory to this pattern of misuse.

Owing to today's family structures with small families and many single-parent households, a lack of basic medical knowledge and experience in the proper assessment of children appears to be another contributing factor to the excessive use of EMTS. Therefore, regular visits to the family pediatrician constitute an opportunity to provide families with relevant medical information in order to cut down on the number of inadequate EMTS use. Notably, we were able to demonstrate that families with more children used EMTS that were not medically indicated less frequently; however, this did not reach statistical significance (*p* = 0.05, OR 1.6, 95%-CI [0.99–2.56]).

Apparently, parents often overestimate the seriousness of their child's disease, which is an important reason for the substantial number of inadequate EMTS use (16, 20). While Camasso-Richardson et al. (9) reported that only 38% of the EMTS occurred during off-hours, about 55% were performed during this time period in our study. This finding is supported by Miller et al. (15) and Seidel et al. (12), who demonstrated a peak in EMTS between 4 and 8 p.m., respectively, noon and 8 p.m. This large number of transports during off-hours may in part be caused by parental subjective assessment that their child may be more severely affected in the evening or at night (21). However, inadequate use of EMTS was not related to day or nighttime in our study.

Miller et al. (15) reported that EMTS use was independent of weekday or season. This is in line with our study, where we did not find any differences with regard to weekdays and seasonal timing on the EMTS use (*p* = 0.37). Although not significant, a higher number of medically not indicated EMTS occurred during January–March (*p* = 0.1). During winter-time, the numbers of non-severe, non-life-threatening pulmonary, and gastrointestinal diseases (bronchitis, gastroenteritis) were higher than during summer months, and might have contributed to this finding. In line with previous reports, the most common diagnoses in our cohort were CNS (30.6%) and pulmonary diseases (14.0%), and traumas (13.2%) (9, 15).

In the study by Camasso-Richardson et al. (9), 40% of families had no other possibility to reach the ED, and 86% (79/92) did not contact their pediatrician/general physician prior to calling the emergency number; 71% (40/56) stated that they could have reached the hospital safely by car, bus or taxi (9, 22). These findings are in line with our results, although the number of persons having no access to a car or other transport possibilities was much lower in our cohort (13.2%). This may have been at least in part related to the specific geographic circumstances in our study. Remarkably and in line with the report by Camasso-Richardson et al. (9), most families in our cohort did not contact any medical provider before calling the EMTS (87.1%). One possible reason for the inadequate EMTS use might have been that by using this service immediate medical help is provided with very short waiting times at the ED as demonstrated by Yarris et al. (22) providing adequate medical information may also reduce the number of inadequate EMTS use in patients with recurrent episodes of medical emergencies, e.g., febrile seizures which were generally categorized as clinical situations, which did not mandate the use of EMTS after the first episode. This may open a window for further parental education.

The decision to use EMTS was at the discretion of the medical dispatcher—a well-trained paramedic with extensive experience in emergency medicine. Thus, in addition to parental educational programs, it appears equally important to provide better training in pediatric emergencies for medical personnel. This notion is corroborated by the fact that assessment of a pediatric emergency in our study was subject to physician's experience in this field, and this is also reflected by the fact that in 19.8% of cases, no agreement between the three evaluating physicians could be reached. However, inter-rater agreement was very good in our study. Moreover, non-medical professions (e.g., teachers) should have a basic medical command of emergency situations in order to initiate adequate lay interventions.

Campaigns to inform the public of a reasonable EMTS use bear the potential to reduce the unnecessary use of EMTS. Ohshige (23) reported a reduced utilization of the ambulance in Yokohama by implementing specific public educational programs. However, by using posters in public facilities as well as handouts and public loudspeaker announcements to inform the population of a responsible EMTS use, patients with serious diseases like stroke and myocardial infarction used the EMTS less frequently (23).

In 2012/2013, 14,263,948 EMTS were performed in Germany causing a financial burden of about 7,38 billion euros (24). Thereof, about 5–10% of all EMTS were pediatric emergencies (11–13), which translates into 700,000–1,400,000 pediatric EMTS and costs of about 369–738 million euros. Projecting our data of inadequate EMTS use (annual extra costs of 114,645 euros) to the national level, our analysis would translate into an approximate estimation of extra costs of about 172–344 million euros per year in Germany. Moreover, when looking at these numbers, it is also important to note that a minor percentage required hospitalizations after initial denial of EMTS (25–27).

Possible shortcomings of our study are related to (a) it being a single-center assessment, and (b) the clinical setting of our study. The University Children's Hospital of Saarland is located in a small town in Germany with a more rural environment. Therefore, our numbers may differ from urban or suburban regions, and our data may not be fully transferable to other regions within Germany or to other industrialized countries. However, the ambulance service of the Saarpfalz region provides EMTS for a number of towns with populations ranging from 50,000–85,0000 people as well.

Moreover, about one third of the patients had to be excluded because parental informed consent could not be obtained for full assessment. This might have influenced the significance of different variables. However, no differences with regard to both demographics as well as the use of EMTS were seen between the two groups (Supplementary Table 1).

We conclude that—as in many other industrialized countries—a substantial number of EMTS use is medically not indicated (4–10, 28). In addition to previous reports, our study provides an analysis of possible contributing risk factors. The inadequate use of EMTS causes substantial extra costs per year. Reasonable EMTS use is needed, requiring continuing multifaceted education of parents, families, and teachers as well as of EMTS staff.

## Data Availability Statement

All datasets generated for this study are included in the article/Supplementary Material.

## Ethics Statement

Full name of the ethics committee that approved the study: Ethics committee of Saarland, Saarbrücken, Germany. Consent procedure used for human participants or for animal owners: This study was carried out in accordance with the recommendations of name of guidelines, name of committee; with written informed consent from all subjects. All subjects gave written informed consent in accordance with the Declaration of Helsinki. The protocol was approved by the name of committee. Any additional considerations of the study in cases where vulnerable populations were involved (for example minors, persons with disabilities or endangered animal species: not applicable.

## Author Contributions

MP was responsible for study design, data analysis, and writing of the manuscript. MB was responsible for study design, data analysis, and critical review of the manuscript. SW was responsible for statistical analysis and critical review of the manuscript. BZ was responsible for data compilation. HS was responsible for data analysis and critical review of the manuscript. MFB was involved in patient care and was responsible for critical review of the manuscript. UG was responsible for critical review of the manuscript. MZ was responsible for critical data analysis and review of the manuscript. SM was chief investigator and responsible for study design, data analysis, and writing of the manuscript.

### Conflict of Interest

The authors declare that the research was conducted in the absence of any commercial or financial relationships that could be construed as a potential conflict of interest.
