# Corrigendum: Assessment of Inadequate Use of Pediatric Emergency Medical Transport Services: The Pediatric Emergency and Ambulance Critical Evaluation (PEACE) Study

**DOI:** 10.3389/fped.2020.583576

**Published:** 2020-09-28

**Authors:** Martin Poryo, Martin Burger, Stefan Wagenpfeil, Bennet Ziegler, Harald Sauer, Marina Flotats-Bastardas, Ulrich Grundmann, Michael Zemlin, Sascha Meyer

**Affiliations:** ^1^Department of Pediatric Cardiology, Saarland University Medical Center, Homburg, Germany; ^2^Medical School, University of Saarland, Homburg, Germany; ^3^Institute for Medical Biometry, Epidemiology and Medical Informatics, Saarland University Medical Center, Homburg, Germany; ^4^SPG, Saarpfalz-Gymnasium, Homburg, Germany; ^5^Department of Neuropediatrics, Saarland University Medical Center, Homburg, Germany; ^6^Department of Anesthesiology, Intensive Care and Pain Therapy, Saarland University Medical Center, Homburg, Germany; ^7^Department of Pediatrics and Neonatology, Saarland University Medical Center, Homburg, Germany

**Keywords:** ambulance, emergency medical transport service, misuse, pediatric emergency, public health

In the original article, there was a mistake in Table 1 as published. **Here, in some cases the wrong numbers were entered**. The corrected Table 1 appears below.

**Table 1:** Affected organ system at admission by ambulance categorized by age.

**Affected organ system**	**≤28 days (*n* = 2)**	**28 days−1 year (*n* = 43)**	**1–12 years (*n* = 186)**	**13–20 years (*n* = 148)**
Central nervous system	0 (0.0%)	7 (16.3%)	65 (34.9%)	44 (29.7%)
Pulmonary	0 (0.0%)	7 (16.3%)	35 (18.8%)	11 (7.4%)
Cardiac	0 (0.0%)	1 (2.3%)	13 (7.0%)	35 (23.6%)
Gastrointestinal	1 (50.0%)	5 (11.6%)	24 (12.9%)	15 (10.1%)
Trauma	1 (50.0%)	14 (32.6%)	32 (17.2%)	3 (2.0%)
Intoxication	0 (0.0%)	2 (4.7%)	8 (4.3%)	29 (19.6%)
Others	0 (0.0%)	7 (16.3%)	9 (4.8%)	11 (7.4%)

*Data are presented as absolute numbers and percentages (brackets). Percentage refers to absolute numbers within the same line*.

The authors apologize for this error and state that this does not change the scientific conclusions of the article in any way. The original article has been updated.

