# Supplementary material for: Assessment of Inadequate Use of Pediatric Emergency Medical Transport Services: The Pediatric Emergency and Ambulance Critical Evaluation (PEACE) Study
Source: Front Pediatr. 2019 Oct 25;7:442. doi: 10.3389/fped.2019.00442 (PMC6823188; doi:10.3389/fped.2019.00442)
Supplement: Supplementary file 2 [file Table_1.DOCX]

Supplementary Material

# Supplementary Tables

**Comparison of the overall cohort and after exclusion of patients without parental informed consent**

| Parameters | Overall cohort including patients without parental informed consent  (n = 518) | Overall cohort only with parental informed consent  (n = 379) | p-value  (2-sided) |
| --- | --- | --- | --- |
| Gender  Male  Female | 275 (53.1 %)  243 (46.9 %) | 211 (55.7 %)  168 (44.3 %) | 0.46 |
| Age  ≤28 days  29 days - 1 year  1 - 12 years  13 - 20 years | 5 (1.0 %)  56 (10.8 %)  257 (49.6 %)  200 (38.6 %) | 2 (0.5 %)  43 (11.3 %)  186 (49.1 %)  148 (39.1 %) | 0.93 |
| Type of referral  Ambulance  Emergency physician | 385 (74.3 %)  133 (25.7 %) | 279 (73.6 %)  100 (26.4 %) | 0.82 |
| Type of treatment  Out-patient  In-patient | 192 (37.1 %)  325 (62.9 %) | 136 (35.9 %)  243 (64.1 %) | 0.96 |
| Time of presentation  8 am - 4 pm  4 pm - 22 pm  22 pm - 8 am | 219 (42.3 %)  163 (31.5 %)  136 (26.3 %) | 171 (45.1 %)  122 (32.2 %)  86 (22.7 %) | 0.46 |
| Weekday of presentation  Workday  Weekend | 342 (66.0 %)  176 (34.0 %) | 254 (67.0 %)  125 (33.0 %) | 0.78 |
| Season  January - March  April - June  July - September  October - December | 188 (36.3 %)  96 (18.5 %)  95 (18.3 %)  139 (26.8 %) | 142 (37.5 %)  78 (20.6 %)  67 (17.7 %)  92 (24.3 %) | 0.73 |
| Rater 1: Indication of EMTS  Yes, indicated  No, not indicated  No, but reasonable  Assessment not possible | 192 (37.1 %)  244 (47.2 %)  69 (13.3 %)  12 (2.3 %) | 147 (38.8 %)  173 (45.6 %)  50 (13.2 %)  9 (2.4 %) | 0.96 |
| Rater 2: Indication of EMTS  Yes, indicated  No, not indicated  No, but reasonable  Assessment not possible | 214 (41.3 %)  188 (36.3 %)  103 (19.9 %)  13 (2.5 %) | 161 (42.5 %)  131 (34.6 %)  77 (20.3 %)  10 (2.6 %) | 0.96 |
| Rater 3: Indication of EMTS  Yes, indicated  No, not indicated  No, but reasonable  Assessment not possible | 227 (43.9 %)  221 (42.7 %)  60 (11.6 %)  9 (1.7 %) | 172 (45.4 %)  154 (40.6 %)  46 (12.1 %)  7 (1.8 %) | 0.94 |
| inter-rater agreement  All 3 raters: Yes, EMTS use was medically indicated  All 3 raters: No, EMTS use was medically indicated | 169 (40.5 %)  248 (59.5 %) | 127 (41.8 %)  177 (58.2 %) | 0.76 |

**Table 6: Comparison of the initial overall cohort including patients without parental informed consent versus the cohort after exclusion of patients without parental informed consent. Data are presented as absolute number and percentages (brackets). Percentage refers to absolute numbers within the same group.**

**Fisher’s exact test if one of the expected cell frequencies was < 5; Chi² test if all the expected cell frequencies were > =5.**

**p-values <0.05 were considered statistical significant**
